# Application of a Molybdenum and Tungsten Disulfide Coating to Improve Tribological Properties of Orthodontic Archwires

**DOI:** 10.3390/nano9050753

**Published:** 2019-05-16

**Authors:** Antonio Gracco, Martina Dandrea, Flavio Deflorian, Caterina Zanella, Alberto De Stefani, Giovanni Bruno, Edoardo Stellini

**Affiliations:** 1 DDS, Faculty of Dentistry, University of Padua, 35100 Padua, Italy; antoniogracco@gmail.com (A.G.); alberto.de.stefani@hotmail.it (A.D.S.); edoardo.stellini@unipd.it (E.S.); 2 DDS, Faculty of Dentistry, University of Rome, 00185 Rome, Italy; martinadandrea@yahoo.it; 3 Department of Industrial Engineering, University of Trento, 38121 Trento, Italy; flavio.deflorian@unitn.it (F.D.); caterina.zanella@unitn.it (C.Z.); 4 Department of materials and manufacturing, School of Engineering, Jönköping University, 553 18 Jönköping, Sweden

**Keywords:** friction, stainless steel archwires, inorganic nanoparticles

## Abstract

Coatings incorporating nanoparticles of molybdenum and tungsten disulfide (MoS_2_ and WS_2_)—known for their lubricating properties—are applied to orthodontic stainless steel wires to verify if there is an improvement in terms of tribological properties during the sliding of the wire along the bracket. To simulate in vitro sliding of the wire along the bracket and evaluate friction 0.019 × 0.025 inches orthodontic stainless steel (SS) wires were subjected to the application, by electrodeposition, of Ni, Ni + MoS_2_, and Ni + WS_2_. The samples produced were analyzed with scanning electron microscopy and assessment of resistance to bending. Thirty-two test conditions have been analyzed, arising from the combination of four types of coatings (SS bare wires and strings with three types of coating), two types of self-ligating bracket (Damon Q, Ormco and In-Ovation R, GAC International), two bracket-wire angles (0° and 5°), two environments (dry and wet). Analyses carried out on the samples show acceptable coatings incorporating MoS_2_ and WS_2_ and a resistance of coatings after a minimum bending. In “dry conditions” a statistically significant decrease in friction occurs for wires coated with MoS_2_ and WS_2_ if associated with the In-Ovation bracket. In “wet conditions” this decrease is observed only in isolated test conditions. Analysis of the wires after sliding tests show little wear of the applied coatings. Nanoparticles are acceptable and similar in their behavior. Improvements in terms of friction are obtained pairing coatings incorporating MoS_2_ and WS_2_ with the In-Ovation bracket in dry conditions.

## 1. Introduction

Orthodontic treatment involves the sliding of a tooth along an orthodontic archwire. Each time this happens, a friction force between archwire and bracket, which is opposed to the movement itself, is generated. For this reason, orthodontic force must exceed this resistance to carry out the displacement. More than 60% of orthodontic force applied to obtain dental movement is expected to be lost due to frictional forces [1], reducing the amount of forces employed by the fixed appliance. Friction reduction would allow the application of a lower orthodontic force, with significant benefits, ranging from a lower risk of root resorption, to the best anchorage control, and reduction of the treatment time [2]. Many years of materials engineering support orthodontic research in finding a solution to this problem. While in the past the focus was mainly on the characteristics of the bracket, these days, however, the most significant progress can be achieved with the application of nanotechnology to the materials science of orthodontic interest. In particular, coating orthodontic archwires with film incorporating nanoparticles of MoS_2_ and WS_2_ seems to be one of the best ways to achieve results in research aimed at the reduction of friction.

In 1985 H.W. Kroto [3,4] discovered a new allotropic form of carbon organized in stable molecules of 60 atoms (C_60_) which was called “fullerene”. The discovery of this molecule gave birth to intensive research for producing other possible structures of carbon. In this way, later, nanotubes (NT) were discovered [5]. The great interest in the unique physical and chemical properties of fullerene-like nanoparticles pushed the research towards the development of inorganic fullerene-like nanoparticles (IF-NP). The attention was focused mainly on chalcogenides (binary salts of S, Se or Te) of the transition metals, in particular on disulfides MoS_2_ and WS_2_, which were described for the first time by Tenne in 1992. These compounds are derived from materials with a layered structure, consisting of a plane of hexagonal crystals (H) of the metal Mo/W intercalated with two floors of anionic sulfur atoms, synthetically 2H-MoS_2_ and 2H-WS_2_, respectively. These structures, potentially unstable, can, in certain conditions, bend and close on themselves, stitching the rim atoms together, becoming similar to graphite and giving birth to particles of 20 to 200 nm [6]. The particular structure of MoS_2_ and WS_2_ nanoparticles makes them excellent solid lubricants, allowing an improvement in friction and wear properties under wet and dry conditions at different load levels [7]. This probably happens due to the penetration of solid lubricant WS_2_ nanoparticles into the interface between rubbed surfaces. As the load between the bodies increases, the nanoparticles gradually deform and exfoliate. In this way the asperities at the interface are coated, allowing a very low shear force sliding motion between the two contacting bodies [6,7,8]. Previous studies of coating stainless steel (SS) or NiTi wires with a metal matrix (nickel-phosphorus, nickel or cobalt) impregnated with IF-WS_2_ nanoparticles showed a significant reduction in friction during in vitro sliding tests [9,10,11,12]. This work aims to reduce friction between orthodontic stainless-steel wires and brackets by coating the wires with self-lubricating nickel (Ni) films containing inorganic amorphous nanoparticles (NP) of tungsten disulfide (WS_2_) and molybdenum disulfide (MoS_2_), which are well-known lubricants.

## 2. Materials and Methods

### 2.1. Nanoparticles

This study was carried out using NP supplied by Nanoshel LLC (Nanoshel LLC, Willmington DE, United States), which has provided the information reported in Table 1.

As regards the advantages brought by the use of these materials, Nanoshel LLC states that a few tenths of a percent in weight of the product can improve tribological properties of the system. When the load between the sliding part is small (low load conditions), friction reduction is mainly ascribable to the bearing-like behavior of NP, that roll between the contact surface, keeping their shape intact. For high load conditions, a coating, induced by the presence of NP, is deposited on the crests of surface roughness and it can reduce direct contact between the asperities, thus, minimize wear.

### 2.2. Bracket

To create a sliding movement, two kind of self-ligating bracket (interactive and passive bracket) were employed:

- 40 In-Ovation (Dentsply Sirona, New York, US) upper right central incisors bracket (tq 12°, tip 5°).
- 40 Damon Q (Ormco, 200 S. Kraemer Blvd., Building E Brea, California 92821) upper right central incisors bracket (tq 15°, tip 5°).

### 2.3. Artificial Saliva

To simulate an intraoral environment, setting up “wet” condition, a saliva substitute (Biotene™ Oral Balance gel) was employed. Since the product is available in a gel formulation, this was diluted with water, having a solution that in a liter of water contained 250 mg of product.

### 2.4. Coatings’ Deposition on Orthodontic Wires

Sixty orthodontic stainless steel (SS) wires with a rectangular cross section (0.019 × 0.025 inches) shape have been subjected to a stage of preparation, consisting in:

- Mechanical abrasion with abrasive paper of silicon carbide (500 grains/unit area), to clean orthodontic wires.
- Solvent degreasing with acetone in an ultrasonic cleaner for 2 min, to remove organic residues and dust of abrasive paper.
- Rinsing with deionized water and drying.
- Positioning of orthodontic wires on a special frame, with the aim of being able to handle more easily without leaving traces on the substrates to be coated.
- Immersion of the frame with the wires in sulfuric acid at 0.5 M with cathodic polarization to −1 V.
- Rinsing with deionized water.

Orthodontic wires were then divided into 4 group, each of these made of 15 wires:

- GROUP 1: control group; wires of this group were not applied any coating.
- GROUP 2: wires of this group were coated with a Ni film by electrochemical co-deposition.
- GROUP 3: wires of this group were coated with a Ni + MoS_2_ film by electrochemical co-deposition.
- GROUP 4: wires of this group were coated with a Ni + WS_2_ film by electrochemical co-deposition.

Orthodontic wires that have to undergo the deposition of a coating (GROUP 2, GROUP 3, GROUP 4) were then inserted in a pre-nickel plating bath (Wood bath) at 25 °C, with a direct current of 0.04 A and starting the process of electrodeposition for 2 min. This procedure allowed the application of a Ni layer to the substrate to promote adhesion of the coating subsequently applied to SS wires.

Afterward, wires of each of the three groups were treated in a different way:

- GROUP 2: wires were inserted in a Watts bath, with a direct current of 0.04 A and starting the process of electrochemical co-deposition for 25 min.
- GROUP 3: wires were inserted in a Watts bath in which 2 g/L of MoS_2_ NP was dispersed; since MoS_2_ NP are hardly wettable, a cationic surfactant (CTAB cetyl trimethyl aminobromide) was added to the bath (0.4 gr/L of CTAB). To minimize agglomeration of MoS_2_ NP, the bath was maintained in constant agitation with the use of a mechanical stirrer (activated 12 h before the deposition and during the entire deposition step) and ultrasound treatment. Electrochemical co-deposition was processed for 25 min with a direct current of 0.04 A.
- GROUP 4: wires were inserted in a Watts bath in which 2 g/L of WS_2_ NP was dispersed; since WS_2_ NP are hardly wettable, a cationic surfactant (CTAB cetyl trimethyl aminobromide) was added to the bath (0.2 gr of CTAB). To minimize agglomeration of WS_2_ NP, the bath was maintained in constant agitation with the use of a mechanical stirrer (activated 12 h before the deposition and during the entire deposition step) and ultrasound treatment. Electrochemical co-deposition was processed for 25 min with a direct current of 0.04 A.

Once the coating process was done, wires were washed with deionized water and dried with compressed air.

### 2.5. Analysis of Coated Orthodontic Wires

The morphology and chemical composition of the film deposited on orthodontic wires were analyzed with scanning electron microscopy (SEM) in association with energy-dispersive X-ray spectrometry (EDS). These analyses were carried out both in surface and in section.

To evaluate the adherence of the coating upon bending of the orthodontic wire (a common clinical procedure) orthodontic wires coated with Ni, Ni + MoS_2_, and Ni + WS_2_ were gradually hand-bent using two spindles, respectively, of 4 mm and 32 mm. The procedure was always performed by the same operator.

### 2.6. Friction Measurements of the Wires

To simulate a sliding movement, a universal device for mechanical measurements (Instron 4502) was employed to carry out friction measurements of the coated and uncoated orthodontic wires. To perform tests, the Damon Q (passive) and In-Ovation (interactive) brackets were employed. Measurements were carried out both in dry and wet conditions, using a saliva substitute (Biotene™ Oral Balance) to simulate the intraoral environment. A dry condition was used to obtain reference values.

A support on which mounting the bracket using the same type of the resin used in orthodontic treatment (Transbond™ PLUS Color Change, 3M Unitek, US) was fixed on the body of the device. The bracket’s support could be rotated varying the angle between bracket and wire to simulate different levels of frictional forces. Two angulations between bracket and wire were chosen: 0° and 5°. The orthodontic wire was fixed above to a 100 N load cell of Instron 4502 and lowered to a weight of 136 g required to hold it taut along the vertical. The temperature was 21 °C.

In this way, the wire engaged the slot of the self-ligating bracket. The load cell was put into motion at a speed of 5 mm/min for a distance of 5 mm. The test began with a steady increase in the force and reached a maximum with the beginning of movement on the wire. This maximum represents the static friction force that is needed to initiate movement.

**Figure 1:**
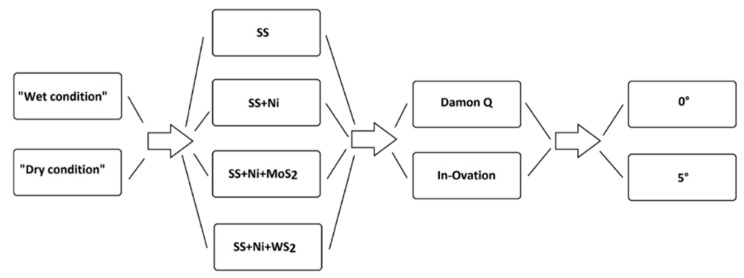
Flow chart of the test condition.

Considering 4 kinds of coatings, 2 types of bracket, 2 types of environment, and 2 different angles, 32 test conditions were created (Figure 1). Each test condition was repeated 5 times. For each repetition, after a run-in period of repeated back and forth movements (5 times) of the wire in the bracket, which is recommended by previous studies [11], 5 values of the frictional force were measured. The data were recorded in the operating system of the Instron and then analyzed and plotted. Data analysis was conducted considering “dry” and “wet” conditions in parallel, independently of one another.

### 2.7. Data Analysis

Descriptive statistic of the frictional forces was accomplished including mean and standard deviation for each of 32 test conditions. Regarding inferential statistic, data were analyzed using a mixed effects model considering coating, bracket, and angle as fixed factors, with wire as a random factor. The multiple comparisons were made with Tukey’s method. Analyses were performed employing R statistical environment (R Core Team, 2015), using the nlme package (Pinheiro & Bates, 2000) for the adaptation of models and lsmeans (Lenth & Hervé, 2015) for multiple comparisons.

### 2.8. Analysis of Coated and Uncoated Orthodontic Wires after Friction Test

Twelve of the 32 test conditions were considered the most significant ones and were analyzed after the Instron test. The morphology and chemical composition of these orthodontic wires were analyzed with scanning electron microscopy (SEM) in association with energy-dispersive X-ray spectrometry (EDS).

## 3. Results

### 3.1. Analysis of Coated Orthodontic Wires

**Figure 2:**
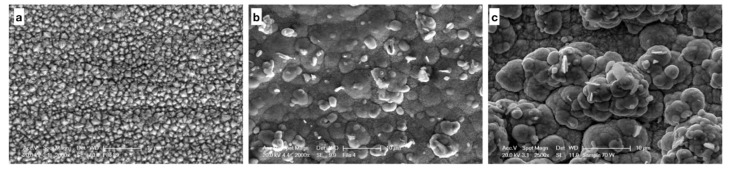
Scanning electron microscopy images (**a**) Ni magnification 2000×, (**b**) Ni + MoS_2_ magnification 2000×, and (**c**) Ni + WS_2_ magnification 2500×.

Scanning electron microscopy analysis shows, for each wires’ group, well-defined continuous coatings. From “in surface” images we can say that coatings’ adhesion is more than good in each of the three groups (Figure 2). Coatings are homogeneous even if the grain is much more evident in the case of films with nanoparticles. Figure 2b,c show the morphology of films that incorporate nanoparticles of MoS_2_, in one case, and WS_2_, in the other, with spherical or cylindrical-like structures.

**Figure 3:**
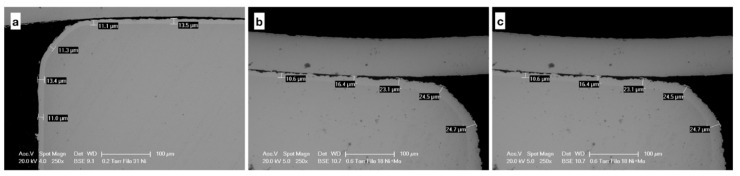
Sectional images of the coating (**a**) Ni, (**b**) Ni + MoS_2_, and (**c**) Ni + WS_2_.

Sectional images show the thickness of the coatings, which is about 10 μm for the wires coated with Ni, 20 μm for those products with Ni + MoS_2_, 15 μm for those with Ni + WS_2_ (Figure 3).

In the case of wires coated with Ni + MoS_2_ and Ni + WS_2_, energy-dispersive x-ray spectrometry (EDS) shows fairly good percentages by weight of MoS_2_ and WS_2_.

**Figure 4:**
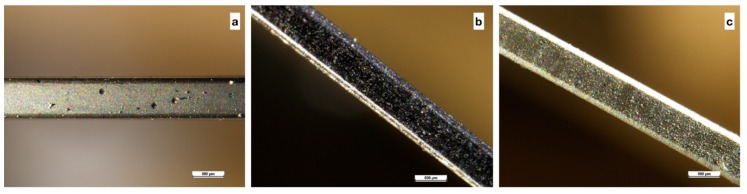
Coating at optical microscope (**a**) Ni, (**b**) Ni + MoS_2_, and (**c**) Ni+WS_2_.

Figure 4 is obtained through an optical microscope and shows three orthodontic wires coated, respectively, with Ni (4a), Ni + MoS_2_ (4b), and Ni + WS_2_ (4c) and subjected to a minimum bending. Overall, no zones of breakage can be found in each type of coatings, maintaining their quality unchanged.

**Figure 5:**
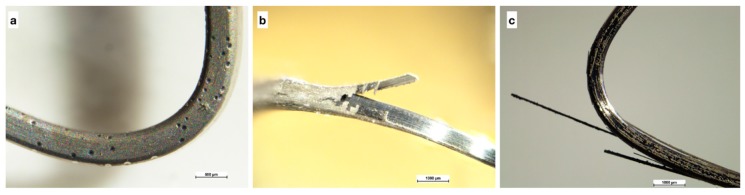
Fractures at optical microscope (**a**) Ni, (**b**) Ni + MoS_2_, and (**c**) Ni + WS_2_.

On the other hand, Figure 5, always obtained through an optical microscope, show damages on Ni + MoS_2_ (5b) and Ni + WS_2_ (5c) coatings as a consequence of maximum bending of the wires, while the wire coated with Ni appears unchanged (5a).

### 3.2. Friction Measurements of the Wires

Data analysis was conducted considering “dry” and “wet” conditions independently of one another, so results will be presented separately.

#### 3.2.1. “Dry” Conditions

For each test condition performed in a dry environment, Table 2 shows the mean and standard deviation of five measurements of the frictional force. From a descriptive point of view, values of the friction coefficient are significantly higher for the wires coated with nickel compared to all other types of wires. Bartlett’s test shows a violation of the assumption of homogeneity of variances between the four coatings (Bartlett’s K^2^ (3) = 160.75, *p* < 0.0001). The adaptation of the mixed effects model showed a significance of all fixed effects included in the model. From the analysis of multiple comparisons performed by Tukey’s method and the average values some conclusions can be drawn in terms of friction coefficient:

- No significant differences were found between Ni + MoS_2_ and Ni + WS_2_ films even if, from a descriptive point of view, wires coated with Ni + MoS_2_ show lower friction values;
- Wires coated with Ni + MoS_2_ and Ni + WS_2_ films have friction values significantly lower than wires coated with Ni;
- If paired to the In-Ovation bracket, SS uncoated wires differ from all others, both from wires coated with Ni (whose friction coefficient is greater) and wires coated with Ni + MoS_2_ and Ni + WS_2_ (whose friction coefficients are lower);
- If paired to the Damon Q bracket, SS uncoated wires have lower friction coefficient only for wires coated with Ni, while they do not significantly differ from wires coated with Ni + MoS_2_ and Ni + WS_2_.

**Figure 6:**
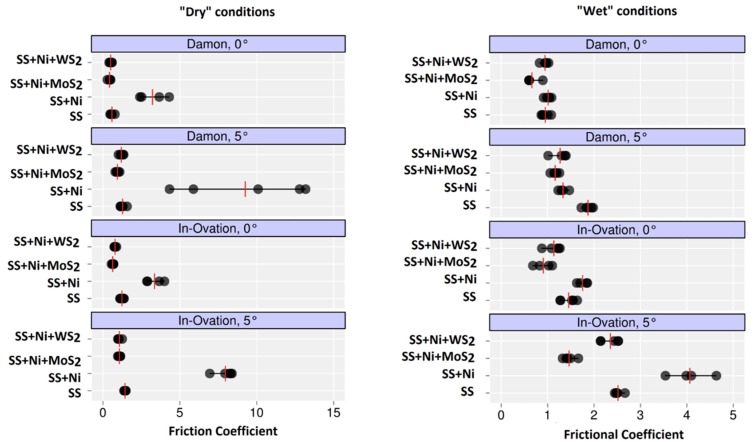
Statistical analysis.

Figure 6 summarizes the results of statistical analysis.

#### 3.2.2. “Wet” Conditions

For each test condition performed in a wet environment, Table 3 shows the mean and standard deviation of five measurements of the frictional force. From a descriptive point of view, values of the friction coefficient are significantly higher for the wires coated with nickel compared to all other types of wires. Bartlett’s test shows a violation of the assumption of homogeneity of variances between the four coatings (Bartlett’s K^2^ (3) = 29.03, *p* < 0.0001). The adaptation of the mixed effects model showed a significance of all fixed effects included in the model. From the analysis of multiple comparisons performed by Tukey’s method and the average values some conclusions can be drawn in terms of friction coefficient:

- Comparing Ni + MoS_2_ and Ni + WS_2_, from a descriptive point of view, wires coated with Ni + MoS_2_ always show lower friction values. Tukey’s test results state that Ni + MoS_2_ presents a lower friction coefficient for both Damon 0° and In-Ovation 5°.
- Wires coated with Ni + MoS_2_ and Ni + WS_2_ films have friction values significantly lower than wires coated with Ni except for Damon 5°.
- If paired to the In-Ovation bracket, SS uncoated wires differ from wires coated with Ni + MoS_2_ (whose friction coefficient is lower).
- If paired to the Damon Q bracket, SS uncoated significantly differ from wires coated with Ni + MoS_2_ and Ni + WS_2_ (whose friction coefficients are lower) only for angulation of 5°.

### 3.3. Analysis of Coated and Uncoated Orthodontic Wires after Friction Test

**Figure 7:**
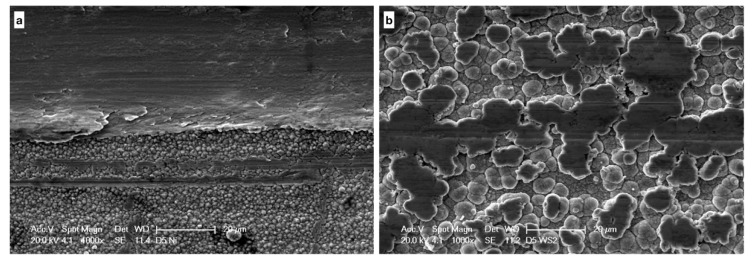
Morphology of damage of the coating material. (**a**) 400× magnification, (**b**) 1000× magnification.

Considering coated and uncoated wires, SEM images have highlighted that the condition with greater wear is “Damon Q–5°”. Wires coated with Ni show a greater amount of damage if compared to the other wires. In contrast, Ni + MoS_2_ and Ni + WS_2_ films seem to have better integrity (Figure 7). Moreover, there is no difference in wear between coatings that incorporate nanoparticles. EDS analysis confirm the presence of nanoparticles in the areas of wear of the wires after friction tests.

## 4. Discussion

From a qualitative point of view, coating stainless steel orthodontic wires with Ni + MoS_2_ and Ni + WS_2_ has provided a homogeneous result. Overall, scanning electron microscopy (SEM) analysis in association with energy-dispersive X-ray spectrometry (EDS) allowed to state that:

- Films’ adhesion was homogeneous, with few uncoated areas.
- The amount of MoS_2_ and WS_2_ in the films is acceptable, confirming that incorporating lubricant compounds within a metallic matrix is one of the best ways to apply a lubricant. This strategy has already been suggested by several studies in the literature [9,10,11,12].
- The coatings’ thickness was contained within values that would allow the insertion of the wires into the slot and the sliding along the bracket.

These results can be considered positive, since the manufacture of the coatings was conducted in the laboratory, and not with industrial production methods.

Good adhesion of Ni + MoS_2_ and Ni + WS_2_ films to SS substrates and limited and constant thickness of the coatings, as evidenced by SEM images, have confirmed the effectiveness of electrodeposition, in agreement with similar trials carried out by other authors [10]. Compared to electroless deposition [9,11], which is frequently used, electrodeposition has made the coatings’ application more “controllable” and manageable, providing consistent repeatability.

Bending orthodontic wires coated with Ni, Ni + MoS_2,_ and Ni + WS_2_ has highlighted the acceptability of the coatings produced for minimum bending, but also the need to improve the resistance to greater bending. A further development may be represented in research for possible treatments that improve the properties of the coatings.

In vitro simulation of clinical conditions that occur with an orthodontic fixed appliance has produced mixed results among themselves. As regards the tests carried out in “dry” conditions, from a descriptive point, there is a decrease in the coefficient of friction for wires coated with Ni + MoS_2_ and Ni + WS_2_ compared to SS wires and Ni-coated wires. Regarding inferential statistic, however, results provide different conclusions. Comparing wires coated with Ni + MoS_2_ and Ni + WS_2_ and SS wires, only testing the In-Ovation bracket, changes in terms of friction coefficient are obtained. This result on the efficiency of Ni + WS_2_ film for the In-Ovation bracket agrees with other researches in the literature [10]. Regarding this, we remind briefly the mechanism for which nanoparticles perform their lubricating action: in low load conditions, friction reduction is primarily due to the “buffer”-like behavior carried out by the nanoparticles, which flow between the surfaces, maintaining their shape; in high load conditions, nanoparticles induce the formation of a deposit at the level of surface roughness, reducing the direct contact between the asperities and minimizing wear. Damon Q behavior (which does not generate statistically significant differences between wires incorporating NP and SS wires) is not comparable with other data from the literature, because similar experiments have not been carried out with the use of such passive self-ligating brackets. Discussing the comparison between Ni + MoS_2_ and Ni + WS_2_ coated wires and Ni coated wires, there was always a statistically significant variation of the friction coefficient, in favor of the sample products incorporating NP. These outcomes for Ni + WS_2_ coating agree with other results in the literature [10]: The improvement in terms of friction coefficient is due to the lubricant behavior of WS_2_, but also to the mechanism of prevention of the formation of an oxide layer deployed by such compounds [10]. In regard to this, a further development of such research may be represented by coating NiTi orthodontic wires to verify if the remarkable properties of MoS_2_ and WS_2_ compounds are confirmed even with these substrates. Comparing Ni + MoS_2_ and Ni + WS_2_ does not lead to statistically significant differences, although from a descriptive point of view Ni + MoS_2_ has values of coefficient of friction lower than Ni + WS_2_. No other studies in the literature compare the lubricating behavior of MoS_2_ and WS_2_ NP in this way.

As regards tests carried out in “wet” conditions, from a descriptive point of view, there is a decrease in friction coefficient for wires coated with Ni + MoS_2_ and Ni + WS_2_ compared to SS wires and Ni-coated wires. Regarding inferential statistics, however, results are extremely heterogeneous with each other. We cannot determine that a particular type of coating provides better results than the others or that the use of one of self-ligating bracket gives better outcomes than the other. A single study in the literature [9] has tested the use of IF-WS_2_ as a lubricant simulating the oral cavity with the use of deionized water: A decrease in the friction coefficient was observed for coated wires. According to the results of this trial and to the limited evidences provided by the literature, further investigations are needed to understand the behavior of Ni + MoS_2_ and Ni + WS_2_ coatings in “wet” conditions.

If we compare SEM images of coated and uncoated wires after friction tests, Ni + MoS_2_ and Ni + WS_2_ coatings show limited areas of damage. This happens both for the minor (wire-slot angle = 0°) and for the major (wire-slot angle = 5°) tribological stress conditions. Chemical composition of Ni + MoS_2_ and Ni + WS_2_ coatings after friction tests remains substantially similar to that obtained with the analysis before Instron measurements.

## 5. Conclusions

This study has allowed to develop coatings qualitatively acceptable: If there is an improvement in the stages of production, in the future they could make a change in orthodontic materials. Positive feedback has occurred using “commercial” NP: In terms of cost/effectiveness they can be considered fairly good, and both disulfides were comparable as regards their characteristics and their behavior. Testing Ni + MoS_2_ and Ni + WS_2_ coated wires with In-Ovation brackets gives better performances in terms of friction than Damon brackets.

The comparison between Ni + MoS_2_ and Ni + WS_2_ coatings did not show any substantial differences: As well as evidenced by sliding tests, SEM images and EDS analysis do not show a better behavior by one coating over the other. Additional investigations should be carried out to establish the behavior of Ni + MoS_2_ and Ni + WS_2_ coatings in conditions that simulate the oral cavity.

## Figures and Tables

**Table 1:** Properties of MoS_2_ and WS_2_.

	MoS_2_	WS_2_
**Purity**	99.90%	99.90%
**Color**	Black	Black
**Morphology**	Spherical	Spherical
**APS**	80–100 nm	40–80 nm
**Density**	5.06 g/cm^3^	ND
**Melting Point**	1185 °C	ND
**Synthesis**	MOCVD	MOCVD

**Table 2:** Mean and standard deviation of five measurements of the frictional force in dry condition.

	SS	SS + Ni	SS + Ni + MoS_2_	SS + Ni + WS_2_
Damon Q/0°	0.58(0.12)	3.22(0.92)	0.42(0.08)	0.50(0.08)
Damon Q/5°	1.27(0.18)	9.25(4.01)	0.94(0.11)	1.19(0.14)
In-Ovation/0°	1.24(0.12)	3.35(0.57)	0.64(0.06)	0.79(0.06)
In-Ovation/5°	1.43(0.06)	7.96(0.59)	1.06(0.07)	1.06(0.13)

**Table 3:** Mean and standard deviation of five measurements of the frictional force in wet condition.

	SS	SS + Ni	SS + Ni + MoS_2_	SS + Ni + WS_2_
Damon Q/0°	0.95(0.09)	1.01(0.07)	0.66(0.13)	0.94(0.07)
Damon Q/5°	1.87(0.10)	1.33(0.10)	1.16(0.07)	1.27(0.18)
In-Ovation/0°	1.45(0.17)	1.75(0.11)	0.91(0.19)	1.13(0.16)
In-Ovation/5°	2.52(0.09)	4.06(0.45)	1.46(0.13)	2.35(0.19)

## References

[1] Kusy R.P., Whitley J.Q. (1997). Friction between different wire-bracket configurations and materials. Semin. Orthod..

[2] Reznikov N., Har-Zion G., Barkana I., Abed Y., Redlich M. (2010). Influence of friction resistance on expression of superelastic properties of initial NiTi wires in “reduced friction” and conventional bracket systems. J. Dent. Biomech..

[3] Taniguchi N. (1974). On the basic concept of nanotechnology. Proceedings of the International Conference Production Engineering.

[4] Kroto H. (2000). C60 and carbon: A postbuckminsterfullerene perspective. Int. J. Mass Spectrom..

[5] Iijima S. (1991). Helical microtubules of graphitic carbon. Nature.

[6] Tenne R., Margulis L., Genut M., Hodes G. (1992). Polyhedral and cylindrical structures of tungsten disulphide. Nature.

[7] Rapoport L., Bilik Y., Feldman Y., Homyonfer M. (1997). Hollow nanoparticles of WS_2_ as potential solid-state lubricants. Nature.

[8] Feldman Y., Wasserman E., Srolovitz D.J., Tenne R. (1995). High-rate, gas-phase growth of MoS_2_ nested inorganic fullerenes and nanotubes. Science.

[9] Katz A., Redlich M., Rapoport L., Wagner H.D., Tenne R. (2006). Self-lubricating coatings containing fullerene-like WS_2_ nanoparticles for orthodontic wires and other possible medical applications. Tribol. Lett..

[10] Redlich M., Gorodnev A., Feldman Y., Kaplan-Ashiri I., Tenne R., Fleischer N., Feuerstein N. (2008). Friction reduction and wear resistance of electro-co-deposited inorganic fullerene-like WS_2_ coating for improved stainless steel orthodontic wires. J. Mater. Res..

[11] Redlich M., Katz A., Rapoport L., Wagner H.D., Feldman Y., Tenne R. (2008). Improved orthodontic stainless steel wires coated with inorganic fullerene-like nanoparticles of WS2 impregnated in electroless nickel-phosphorous film. Dent. Mater..

[12] Samorodnitzky G.R., Redlich M., Rapoport L., Feldman Y., Tenne R. (2009). Inorganic fullerene-like tungsten disulfide nanocoating for friction reduction of nickel—Titanium alloys. Nanomedicine.

